# Physical Activity and Purkinje Cells: Mechanisms of Cerebellar Plasticity and Neuroprotection

**DOI:** 10.3390/jfmk11030307

**Published:** 2026-08-03

**Authors:** Nicoletta Palmeri, Agata Grazia D’Amico, Daniela Maria Rasà, Carla Cavallaro, Giuseppe Musumeci, Velia D’Agata, Grazia Maugeri

**Affiliations:** 1 Section of Anatomy, Histology and Movement Sciences, Department of Biomedical and Biotechnological Sciences, University of Catania, 95123 Catania, Italy; nicoletta.palmeri@unict.it (N.P.); carlacavallaro2000@gmail.com (C.C.); g.musumeci@unict.it (G.M.); vdagata@unict.it (V.D.); 2 Department of Drug Sciences, University of Catania, 95123 Catania, Italy; agata.damico@unict.it; 3 Department of Medicine and Surgery, Kore University of Enna, 94100 Enna, Italy; danielamaria.rasa@unikore.it

**Keywords:** Purkinje cells, physical activity, cerebellum, movement, coordination, cognitive functions

## Abstract

The cerebellum plays a key role in regulating movement, coordination, and certain cognitive functions. Purkinje cells are the principal neurons of the cerebellum, essential for integrating information and modulating output to other parts of the central nervous system. Physical exercise is crucial for maintaining psychophysical well-being and improving quality of life. Moreover, its beneficial effects on brain health are well established. After a brief overview of cerebellar anatomy, this narrative review aims to summarize current experimental evidence on the effects of physical activity on Purkinje cells, particularly in the context of neuronal plasticity and neuroprotection. Special attention is given to structural and functional adaptations under both physiological and pathological conditions, including neurodegenerative diseases, neurodevelopmental disorders, traumatic brain injury, and aging. Overall, the available findings suggest that Purkinje cells represent a key cellular target of exercise-induced cerebellar plasticity. Physical activity may therefore contribute to preserving cerebellar function and promoting neuroprotection through mechanisms involving structural remodeling and synaptic adaptation of Purkinje cells.

## 1. Introduction

Brain health is fundamental for longevity, quality of life, and the preservation of cognitive function during aging, as well as the risk reduction of neurodegenerative diseases [1,2]. Numerous factors contribute to brain health; regular physical activity has grown as one of the most effective, accessible, and scalable non-pharmacological strategies to prevent cognitive decline, promote neurological well-being [2,3,4].

In particular, regular physical activity improves synaptic plasticity, neurogenesis, and cerebral perfusion, inducing structural and functional brain adaptation [3,4]. These promote neuroplasticity, improve quality of life [2] and support counteracting age-related cognitive decline even in healthy older adults and with cognitive impairment [3,4].

At the biological level, exercise induces multiple beneficial effects on central nervous system, increasing hippocampal neurogenesis, modulation of cerebral metabolism, angiogenesis, and improved cerebral blood flow [4,5]. In particular, the hippocampus represents one of the main regions involved in the effects of exercise: experimental studies have shown that physical activity enhances neuronal survival in the dentate gyrus, as well as improving synaptic plasticity [6,7,8,9,10,11,12].

Physical activity has also been shown to exert neuroprotective effects against several neurodegenerative disorders, such as Alzheimer’s, Parkinson’s, and Huntington’s diseases; amyotrophic lateral sclerosis; and multiple sclerosis, by delaying onset and slowing the functional decline associated with these conditions [13,14,15,16,17,18,19]. These benefits are related to increased neurogenesis, increased neuronal survival, and reduced inflammation [8,10,16,20,21]. Additionally, therapeutic exercise programs showed to improve functional recovery after stroke [22]. Meanwhile, physical activity exerts positive effects on mental well-being, reducing depression symptoms and anxiety and improving quality of sleep and life [23,24,25]. These effects are mediated by modulation of neurotransmitters, such as dopamine and serotonin, and by reductions in oxidative stress and neuroinflammation (suppressing the activation of microglia and astrocytes) [5,26,27,28].

Exercise contributes to cognitive resilience throughout life, promoting neuroplasticity, that is, the brain’s ability to adapt to new experiences and recover from any damage [29,30,31]. In this contest, neurotrophic factors play a central role: brain-derived neurotrophic factor (BDNF), vascular endothelial growth factor (VEGF), and insulin-like growth factor 1 (IGF-1) act in a coordinated manner to promote neurogenesis, synaptic plasticity, and angiogenesis [32,33,34,35].

Neuroimaging evidence confirms that aerobic activity changes brain structure and function, improving memory, attention, executive function, and processing speed [26]. These effects induce the improvement of hippocampal volume, cortical thickness, white matter integrity [36,37,38].

Both aerobic and anaerobic exercise contribute to brain health, although with partly different effects. Aerobic exercise is associated with more noticeable improvements in cognitive function and brain structure, including increases in gray matter volume and neuronal connectivity. Anaerobic exercise, on the other hand, is particularly effective in improving motor control and some aspects of cognitive function, such as reaction time [39,40,41]. Hormonally, anaerobic exercise elevates levels of insulin-like growth factor 1 (IGF-1) and testosterone, both of which are crucially involved in synaptic plasticity and neurogenesis [42,43]. In particular, testosterone combats age-related cognitive decline [14], while both hormones enhance mental well-being and mitigate stress [44]. Furthermore, neuroprotective effects of exercise depend on specific parameters, such as frequency, duration, and intensity. Regular, moderate-intensity physical activity (three times a week for approximately 40 min) has been shown significant benefits for brain health [4,45].

Synaptic plasticity is the primary cellular mechanism through which physical activity promotes functional recovery and cognitive adaptability. In the central nervous system, this plasticity is traditionally assessed through two opposing processes: long-term potentiation (LTP) and long-term depression (LTD). While classical cerebellar learning relies largely on LTD at the synapse between parallel fibers and Purkinje cells to recalibrate motor commands, pioneering studies have converged to demonstrate that both voluntary and forced exercise protocols dramatically increase synaptic efficiency and alter neuronal cytoarchitecture. For example, voluntary wheel running has been shown to double the number of surviving neonatal cells and increase net plasticity in adult rodent models [6]. This activity-induced remodeling directly alters the density and arborization of dendritic spines in the hippocampus, entorhinal cortex, and dentate gyrus, altering the ratio and proportions of mature to immature spines [46,47]. Neurophysiologically, forced treadmill running directly enhances the induction and maintenance of LTP within the dentate gyrus by upregulating BDNF and altering synaptic thresholds [48]. By transposing these well-established mechanisms to the cerebellar cortex, exercise closely mirrors these cortical and hippocampal dynamics. Exercise-dependent increases in neurotrophic factors and calcium (Ca^2+^) dynamics modulate downstream kinases and phosphatases that regulate both LTD and intrinsic LTP in Purkinje cells, thereby improving motor coordination, learning calibration, and providing a robust neuroprotective shield against apoptotic degradation under pathological conditions.

Consequently, mice engaged in physical activity during the late postnatal period exhibited Purkinje cells with larger dendritic trees and higher spine density compared to their sedentary littermates, suggesting that early motor activity may influence Purkinje cell maturation and contribute to improved motor coordination [42].

Overall, evidence indicates that exercise represents an effective non-pharmacological strategy for promoting brain health. Through modulation of molecular, cellular and systemic mechanisms, physical activity improves cognitive function, supports mental well-being and contributes to the prevention of cognitive decline and neurodegenerative diseases [5,49,50]. Finally, the benefits of exercise are observed throughout life, improving cognitive development in young people and preserving cognitive function in older adults [2,51].

Although the benefits of physical exercise on brain health are widely documented, the current literature has focused almost exclusively on the adaptive responses of the cortex and hippocampus. Consequently, the cerebellum has long been overlooked, leaving a significant gap in our understanding of how physical activity modulates its specific neuronal networks. A dedicated focus on Purkinje cells is crucial, as they represent the sole output pathway of the cerebellar cortex and are uniquely vulnerable to neurodegenerative and metabolic insults. Therefore, gathering and critically analyzing current experimental evidence focused on Purkinje cells allows for us to redefine the role of the cerebellum in exercise.

## 2. Methods

This narrative review includes the recent literature to provide a balanced and comprehensive overview of innovative discoveries regarding the effects of physical activity on Purkinje cells. Particular emphasis is placed on neuronal plasticity and neuroprotection under both physiological and pathological conditions, such as Alzheimer’s disease, Parkinson’s disease, neurodevelopmental disorders, and traumatic brain injury. Subsequently, the selected articles are discussed in the following subsections: “Neuroprotective Effects in Alzheimer’s and Parkinson’s Disease Models”, “Modulation in Neurodevelopmental and Neurobehavioral Disorders”, ‘’ Neurotrophic Signaling and Plasticity Markers in Healthy Adulthood’’ and “Effects under Aging, Traumatic Injury, and Hypoperfusion Conditions”.

Keywords for the literature search included physical activity, exercise, cerebellum, Purkinje cells, and neuroprotection. The search was limited to studies published in English. Protocols, abstracts without a full-text article, and papers replicating data from other studies were excluded. The literature search was conducted from December 2025 to June 2026 across PubMed, Scopus, Web of Science, and Google Scholar. Ultimately, 16 sources met the eligibility criteria and were deemed appropriate for the purpose of this review. Since an insufficient number of original articles was available to perform a systematic review, a narrative scoping review approach was preferred.

## 3. Cerebellum: Anatomy and Functions

The cerebellum is located in the posterior portion of the brain, inferior to the occipital cortex and dorsally to the pons and medulla oblongata. It is separated from the overlying brain by the tentorium cerebelli and is connected to the brainstem by three pairs of cerebellar pedicles that contain afferent and efferent pathways and establish connections with numerous central nervous system structures [52].

Cerebellar afferent pathways transit primarily through the inferior and middle cerebellar peduncles. Instead, efferent pathways leave the cerebellum through the superior cerebellar peduncle [53], representing the main cerebellar exit route towards the overlying structures. The superior cerebellar peduncle consists predominantly of efferent fibers originating from the deep cerebellar nuclei (DNC), dentate, interposed, and fastigial, although it also receives some afferents, including those from the ventral spinocerebellar tract [54].

Instead, the middle cerebellar peduncle establishes a direct connection with the pons and carries mainly pontocerebellar fibers from the contralateral pontine nuclei, which convey information from the cerebral cortex to the cerebellum [55]. The inferior cerebellar peduncle receives afferents from the vestibular nuclei, spinal cord, and brainstem tegmentum and transmits efferent to the vestibular nuclei and reticular formation.

Functionally, the cerebellum integrates afferent inputs within the cerebellar cortex and deep cerebellar nuclei, which also receive direct collateral forecast. These inputs are transmitted mainly by mossy fibers originating from spinal cord, several brainstem nuclei, and the cerebral cortex (including motor and sensory area) [56].

The main cerebellar afferents are excitatory mossy climbing fibers. Mossy fibers originate from the pontine nuclei and made synapse with granule cells in the cerebellar cortex. Instead, climbing fibers originate exclusively from the inferior olivary nucleus and form direct synapses with Purkinje cells and deep cerebellar nuclei [57].

The cerebellar cortex sends its output signals exclusively through Purkinje cells, which inhibit [52,53,58]; the deep cerebellar nuclei and modulates cerebellar output directed to other structures of the nervous system.

Morphologically, the cerebellum features an extremely folded surface organized into large lobules, which are further divided into numerous thin ridges called folia. This highly laminated architecture remarkably expands the cerebellar cortical surface [59]. Each folium consists of an outer layer of highly folded gray matter (the cerebellar cortex) sur-rounding a central white matter core. The white matter, forming the corpus medullare, spreads deeply into the lobules and individual cortical folia [52].

The cerebellar cortex is organized into three distinct layers: the inner granular layer, the Purkinje cell layer, and the outer molecular layer.

The molecular layer contains stellate cells and basket cells, in addition to granule cell axons and Purkinje cell dendrites [60]. Many cerebellar cortical neurons perform inhibitory functions, including Purkinje cells, Golgi cells, stellate cells, and basket cells. In contrast, granule cells and unipolar brush cells are excitatory [60].

The middle layer consists of Purkinje cell bodies and Bergmann cells, representing the exit layer of the cerebellar cortex [52].

The inner granular layer contains the cell bodies of granular cells, Golgi cells, Lugaro cells, unipolar brush cells and axons of Purkinje cells, representing the main information entry region into the cerebellar cortex [52]. Granule cells, the most abundant neuronal population in the brain, send axons into the molecular layer, where they bifurcate into parallel fibers that form excitatory synapses with Purkinje cell dendrites and activate inhibitory interneurons.

The integrated activity of these cells regulates signaling directed to the deep cerebellar nuclei. Information reaching the cerebellum via mossy fibers is processed in the granular layer and transmitted to Purkinje cells, while collaterals of these pathways directly activate the deep cerebellar nuclei. Purkinje cells, exerting an inhibitory action on these nuclei, then form a modulator circuit that controls cerebellar output.

The cerebellar white matter is highly branched and houses four pairs of deep cerebellar nuclei: the fastigial nucleus; the interposed nuclei, consisting of the globose and emboliform nuclei; and the dentate nuclei, which represent the largest nuclei [52]. These nuclei are the main route of information out of the cerebellum to the rest of the brain.

Macroscopically, the cerebellum is divided into two lateral hemispheres separated by midline vermis. The vermis is mainly involved in the control of the axial musculature, whereas the lateral hemispheres contribute to the planning and execution of limb movements. Each hemisphere is further divided into three main lobes, separated by the primary and posterolateral fissures, and further divided into numerous lobules by additional sulci [52,61].

Functionally, the cerebellum is divided into three main domains, partially overlap with anatomical subdivision: the vestibulocerebellum, the spinocerebellum, and the cerebrocerebellum.

The vestibulocerebellum, the phylogenetically oldest portion, corresponds mainly to the flocculonodular lobe. It receives vestibular inputs and regulates balance, eye movements, and postural stability through connections with the vestibular nuclei; these inputs are processed and sent back to the nuclei and the spinal cord [52,53,58,60].

The spinocerebellum mainly includes the anterior lobe, the vermis, and the regions associated with the fastigial and interposed nuclei. This region receives proprioceptive information from the dorsal column of the spinal cord and sends back signals to fine-tune the execution of the movement.

Finally, the cerebrocerebellum, phylogenetically the most recent portion of the cerebellum, consists mainly of the lateral regions of the cerebellar hemispheres. It receives information from the cerebral cortex via the pontine nuclei and sends projections to the thalamus and red nucleus, interacting with the motor cortex for planning and programming voluntary movements.

## 4. Focus on Purkinje Cells

Purkinje cells (PCs) were first described by Johan Evangelista Purkinje in 1837 [62]. They were subsequently studied in detail by Camillo Golgi in 1882 and by Santiago Ramón y Cajal, who in 1888 identified the presence of dendritic spines along the extended lotus dendritic tree [63].

Purkinje cells represent one of the major neuronal types of the cerebellar cortex and constitute the largest and most complex cells in the entire central nervous system. They are located in the middle layer of the cerebellar cortex, where they arrange themselves neatly into a single cell layer, called the Purkinje cell layer.

Functionally, Purkinje cells represent the central element of the cerebellar circuit [64]. In fact, they represent the only efferent pathway of the cerebellar cortex, allowing for it to integrate incoming sensory and motor information, generating the entire cortical output directed to the deep cerebellar nuclei and other structures of the central nervous system [65].

**Figure 1:**
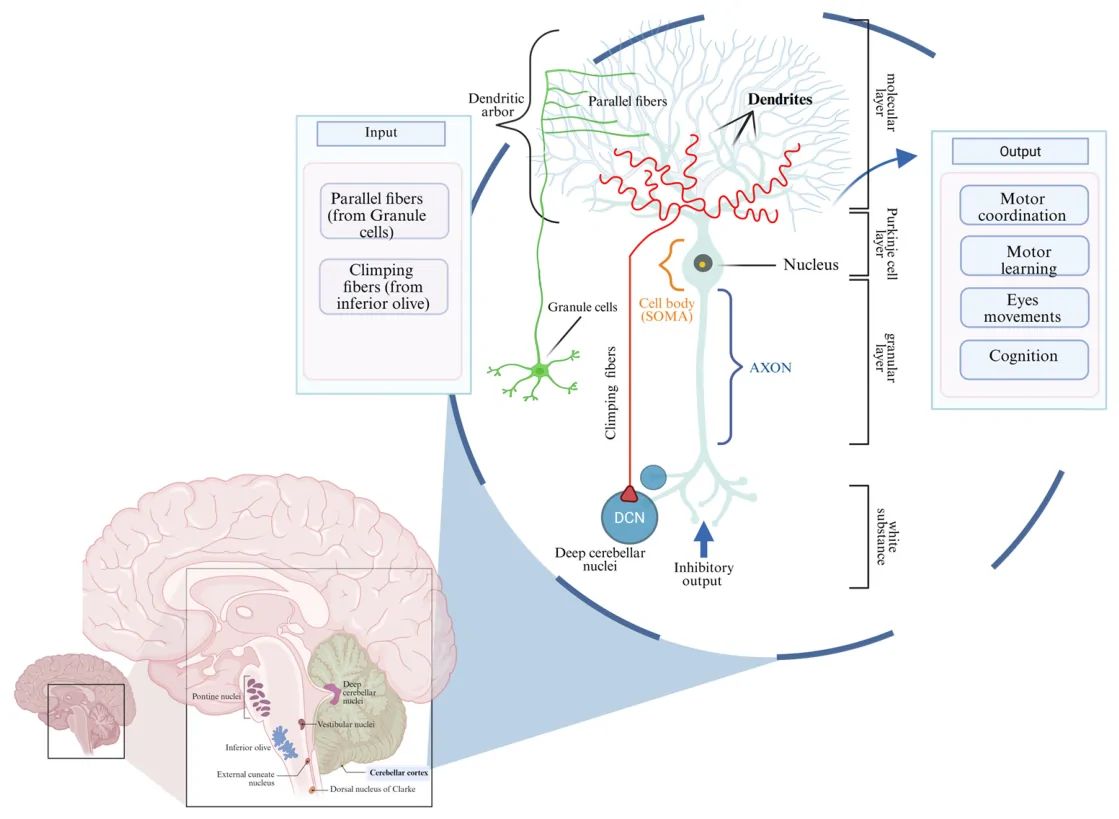
Morphological and functional organization of Purkinje cells. Purkinje cells represent the only exit neurons in the cerebellar cortex and integrate excitatory synaptic inputs from parallel fibers from granule cells and climbing fibers from the lower olive. These neurons contribute to motor coordination, motor learning, posture regulation, eye movement control, and higher-order cognitive processing through inhibitory projections to the deep cerebellar nuclei. Created in https://BioRender.com. Access date: 13 April 2026.

Morphologically, Purkinje cells possess a large soma, or cell body, from which originates an extensive, highly branched dendritic tree that develops into the overlying molecular layer. This complex dendritic arborization allows for a single Purkinje cell to receive and integrate an extremely large number of synaptic inputs from different sources. Synaptic currents generated along dendrites are channeled to the axon initial segment (AIS), where the action potential originates [66]. The axon of Purkinje cells is generally single, thin, and sparsely branched, but establishes numerous synaptic contacts with target neurons, particularly in the deep cerebellar nuclei. Purkinje cells exert a predominantly GABAergic inhibitory action on these neurons, contributing to the fine regulation of motor commands and motor learning processes. Purkinje cells receive two main types of excitatory synaptic inputs: from parallel fibers and climbing fibers. Parallel fibers arise from the axons of granule cells and run longitudinally in the molecular layer, establishing numerous synapses with the dendrites of Purkinje cells. Through these connections, it allows for each Purkinje cell to integrate information from a large number of afferent sources [67]. These fibers transmit information about sensory inputs related to position, body movements, and planned motor programs. In contrast, each Purkinje cell receives input from a single climbing fiber originating from neurons in the lower brainstem olive. These fibers establish extremely powerful and selective synaptic connections, wrapping around the proximal dendrites of the target cell and generating an intense excitatory response. Climbing fibers carry signals of motor error associated with discrepancies between predicted and executed movement (i.e., when a movement does not go as expected). These play an essential role in motor adaptation, allowing for real-time correction of movements (Figure 1).

Purkinje cells are GABAergic neurons, involved in the regulation of movement, motor coordination, and some cognitive functions [68,69,70]. They also play a central role in motor control, sensory processing, and motor learning processes [71,72,73].

Due to their strategic location as the sole output of the cerebellar cortex, Purkinje cells play a critical role in motor coordination by modulating the activity of the deep cerebellar nuclei. Through this modulation, they help refine the commands sent to the muscles and regulate the strength, direction, and timing of movements, contributing to the precise execution of motor commands; this ensures that our movements are not merely intentional.

A central aspect of Purkinje cell function concerns their ability to integrate sensory information from different regions of the central nervous system. They make it through their large dendritic trees. The interaction between inputs from parallel fibers and climbing fibers allows for the cerebellum to continuously adapt motor activity based on sensory feedback, ensuring smooth and accurate movements [74].

Purkinje cells also show marked synaptic plasticity, which represents one of the main cellular mechanisms underlying motor learning. Motor learning is a fundamental aspect of our daily activities, allowing for us to acquire new skills, adapt to changes, and improve our performance over time, and often requires trial and error. During the acquisition of new skills, error signals transmitted by climbing fibers induce changes in Purkinje cell activity, resulting in adaptive changes in motor commands and progressively improving performance [75].

Traditionally, instructive signals of cerebellar learning have been attributed primarily to climbing fibers. However, more recent studies suggest that Purkinje cell activity may also directly contribute to motor learning processes. In particular, Nguyen-Vu et al. [76] demonstrated that via selective optogenetic stimulation, flocculus Purkinje cell activity is sufficient to guide associative motor learning in vivo during vestibulo-ocular reflex (VOR) adaptation even in the absence of visual cues [77,78].

Consistent with these observations, neurophysiological recording studies in awake animals have shown that Purkinje cells dynamically modify their activity during sensorimotor calibration tasks. These findings suggest that they not only represent the final output of the cerebellar cortex, but actively participate in modulating motor command, transmitting instructive signals to the deep cerebellar nuclei, and predicting the kinematic consequences of movement, with a role that varies at different stages of motor learning [79].

Purkinje cells also play an important role in controlling balance and posture through the integration of information from the vestibular system. By processing information about the body’s spatial orientation, they rapidly modulate the response of postural muscles, allowing for immediate adaptation to sudden changes in position.

A further relevant contribution concerns the control of eye movements. Purkinje cells participate in the regulation of saccades and the slow pursuit of moving objects, integrating information relating to the direction and speed of moving objects and coordinating, so as to maintain concentration on the object [80].

Structural or functional alterations of Purkinje cells are associated with both motor disorders and non-motor neurological conditions. In particular, their degeneration is a key element in the pathogenesis of cerebellar ataxia [81,82]. Purkinje cells are particularly vulnerable to ischemic damage [83]. Cell death observed under these conditions (following cerebellar ischemia) has been associated with excitotoxicity mechanisms mediated by glutamate release and increased intracellular calcium [84]. Further evidence indicates that Purkinje cell loss has been observed in several neurological conditions, including Alzheimer’s disease and attention deficit/hyperactivity disorder (ADHD) [72].

## 5. Cerebellar Responses to Exercise: Specific Effects on Purkinje Cells

Physical activity is a powerful modulator of central nervous system homeostasis, contributing significantly to brain well-being and driving structural and functional neuronal plasticity.

Traditionally, the cerebellum has been considered a structure primarily involved in controlling voluntary movements, motor coordination, balance, and gait regulation [85]. Together with the cerebral cortex and basal ganglia, it plays a critical role in producing smooth, continuous movements, as well as processing and correcting motor errors [86].

In recent decades, however, anatomical and neuroimaging evidence has progressively expanded this view, demonstrating that the cerebellum also participates in higher-order functions and different cognitive processes [87,88,89,90]. Currently, it is considered an integrative node within a distributed network that connects sensory, motor, cognitive, affective and social functions [91,92,93,94].

Within the cerebellar cortex, Purkinje cells represent the sole output of the cerebellar cortex, playing a fundamental role not only in motor coordination and balance but also in complex cognitive processes. In recent years, a growing body of experimental evidence, primarily derived from murine models subjected to variable treadmill exercise protocols, has highlighted the remarkable sensitivity of these neurons to physical exercise and training.

**Figure 2:**
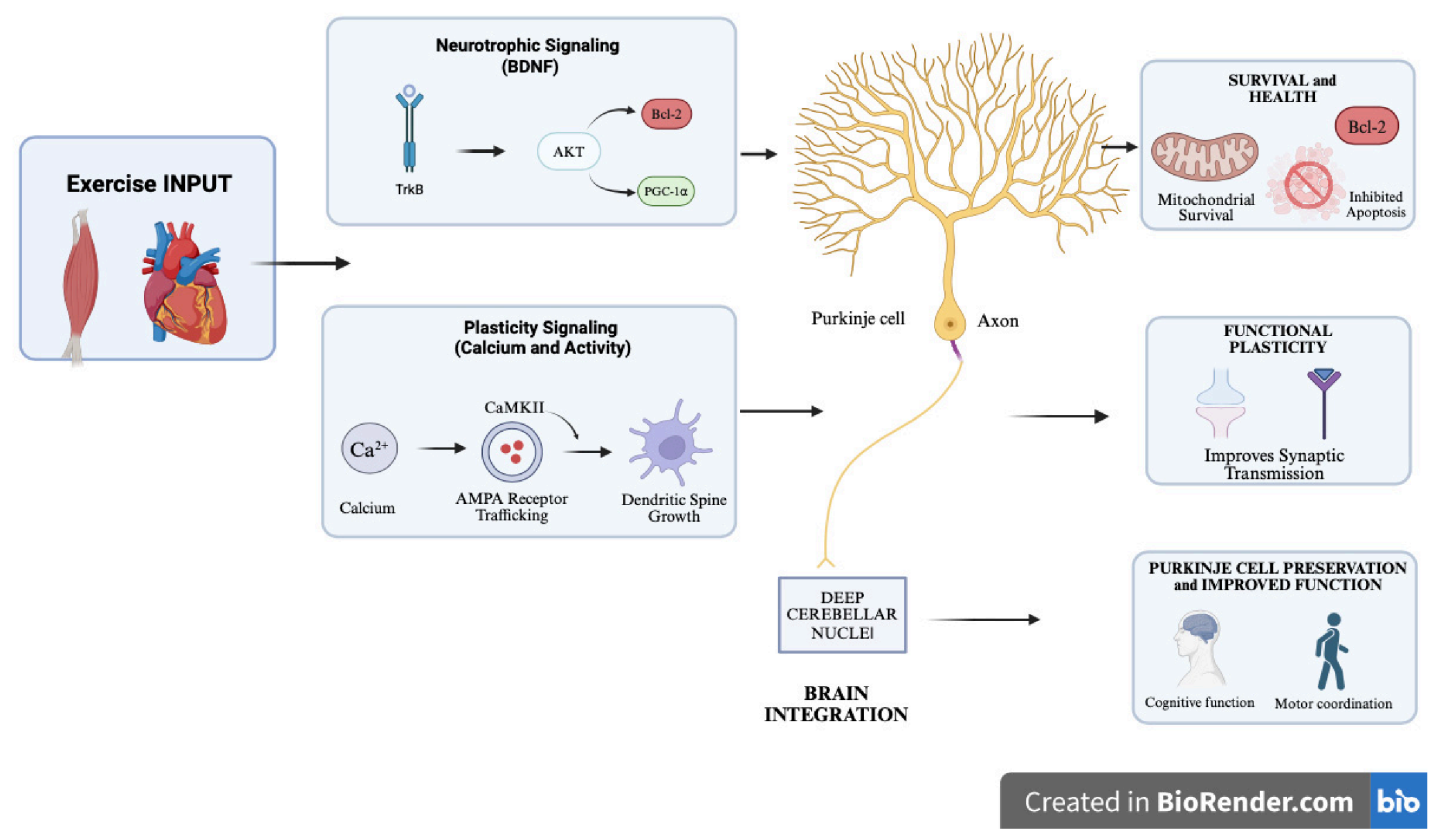
Proposed molecular mechanisms underlying exercise-induced Purkinje cell survival and synaptic plasticity. Physical exercise triggers systemic inputs that activate neurotrophic and calcium-dependent signaling cascades within Purkinje cells. The BDNF/TrkB axis activates AKT, promoting mitochondrial survival via PGC-1α and anti-apoptotic signaling via Bcl-2. Concurrently, exercise-driven calcium (Ca^2+^) dynamics and CaMKII activation enhance AMPA receptor trafficking and dendritic spine growth, leading to improved synaptic transmission. Through efferent projections to deep cerebellar nuclei, these cellular adaptations support overall Purkinje cell preservation, contributing to enhanced motor coordination and cognitive function under physiological and pathological conditions. Created in https://BioRender.com. Access date: 20 July 2026.

Mice engaged in physical activity during the late postnatal period exhibited Purkinje cells with larger dendritic trees and higher spine density compared to their sedentary littermates, suggesting that early motor activity may influence Purkinje cell maturation and contribute to improved motor coordination [95]. Overall, physical exercise activates key neurotrophic cascades and calcium-dependent plasticity signaling, promoting Purkinje cell preservation and functional integration (Figure 2).

### 5.1. Neuroprotective Effects in Alzheimer’s and Parkinson’s Disease Models

In the context of Alzheimer’s disease (AD), treadmill exercise has shown neuroprotective effects on Purkinje cells. In a rat model with intracerebroventricular (ICV) administration of Aβ [25,26,27,28,29,30,31,32,33,34,35], Lee et al., observed that a 4-week treadmill exercise protocol, initiated two days post-injection, improved motor coordination and balance. This functional recovery was associated with attenuated Purkinje cell loss and the suppression of reactive astrogliosis in the cerebellum [96]. Consistently, Jin et al., demonstrated that a longer, 12-week treadmill training program in triple-transgenic (3xTg-AD) mice reduced the accumulation of extracellular β-amyloid and phosphorylated tau, mitigated mitochondrial dysfunction, and enhanced Purkinje cell survival. Taken together, these findings suggest a robust protective effect of physical exercise against the progression of cerebellar pathology in AD [97].

Similar neuroprotective effects have also been observed in models of Parkinson’s disease (PD). This disease shows symptoms such as slowness of movement, tremors at rest, stiffness, gait disturbance, and postural instability. In rats treated with rotenone (a more prevalent neurotoxin for producing animal models for PD), Lee et al. demonstrated that treadmill running for 30 min once a day for 4 weeks, starting 4 weeks after birth, improved motor balance and coordination, reduced Purkinje cell loss in the cerebellar vermis, and inhibited the activation of reactive astrocytes and microglia, contributing to the reduction in apoptotic processes of cerebellar Purkinje cells [98]. Another study investigated the effects of aerobic exercises on Purkinje cells of cerebellum in Parkinson’s rats with fetal stress [58]. The results showed that perinatal stress, induced by immobilization stress from day 8 to 21 for 3 h each day, significantly decreased the number of Purkinje cells. Aerobic training consisting in treadmill five days a week for 8 weeks, showed a significant increase in the number of Purkinje cells [99].

### 5.2. Modulation in Neurodevelopmental and Neurobehavioral Disorders

Similar benefits have also been observed in neurodevelopmental disorders. Autism is a complex developmental disorder with impairment in social interaction, communication, repetitive behavior, and motor skills. Specifically, in the valproic acid (VPA)-induced model of autism, Cho et al. reported that four weeks of treadmill exercise improved motor dysfunction and increased Purkinje cell survival by reducing astrocytic and microglial activation in autistic rat pups [100]. Recently, Shaban et al. [101] investigated the effects of endurance training in valproic acid-treated mice. In particular, mice received VPA at the fourteenth postnatal day. Then, they were subjected to treadmill training 5 days/week for 6 weeks. Results showed a significant increase in the quantity of Purkinje cell in the autistic trained mice. Moreover, Purkinje neurons were more likely to survive when the autistic mice engaged in treadmill activity because it inhibited reactive astrocytes and microglia [101].

### 5.3. Neurotrophic Signaling and Plasticity Markers in Healthy Adulthood

Moderate physical activity has been shown to modulate the expression of Activity-Dependent Neuroprotective Protein (ADNP), a factor involved in brain development and neuronal plasticity. Recently, Maugeri et al. reported increased expression of ADNP and β-tubulin III within the cerebellar Purkinje cells and dentate gyrus of rats subjected to a 12-week moderate physical activity protocol (treadmill training for 20 min daily, 5 days per week). Notably, the authors observed an enhanced cytoplasmic distribution of ADNP, which colocalized with β-tubulin III in the Purkinje cells of exercised rats compared to sedentary controls [102]. Further evidence indicates that moderate physical activity may modulate cerebellar neurotrophic signaling. Specifically, recent data demonstrate that a 12-week moderate exercise protocol increases the expression of pituitary adenylate cyclase-activating polypeptide (PACAP) and its specific receptor, PAC1R, in the cerebellar cortex. This upregulation is accompanied by an elevated co-expression of the immature neuron marker doublecortin (DCX) within both Purkinje and granule cells, suggesting a potential involvement of physical activity in modulating neuronal plasticity and cell survival pathways [103]. Even in healthy adult rats, 4-week exercise is able to induce structural changes in Purkinje cells. González-Burgos et al. observed an increase in the density and proportions of dendritic spines in the simple lobule of the cerebellum after both moderate and intense treadmill training, indicating a plastic adaptation of Purkinje cells associated with motor learning [104].

Similar neuroprotective effects have been observed in young rats subjected to chronic training. In a study by Huang et al., six-week-old male rats underwent one week of treadmill acclimation before being divided into sedentary and exercised groups; the latter was then subjected to eight weeks of moderate-intensity training. The authors demonstrated that prolonged moderate exercise improved motor performance and increased both the dendritic volume and spine density of Purkinje cells. Furthermore, this structural plasticity rendered the Purkinje cells less vulnerable to immunotoxic damage induced by the intraventricular administration of OX7-saporin. Consequently, because these cells exhibited relative resistance to the immunotoxin, the trained rats maintained stable motor performance even under toxin-treated conditions [105].

### 5.4. Effects Under Aging, Traumatic Injury, and Hypoperfusion Conditions

The neuroprotective effect of physical activity is also evident during aging. Stereological studies by Larsen et al. demonstrated that sedentary aged rats exhibited a significant reduction in the number and volume of Purkinje cell soma compared to trained aged rats, which showed values comparable to those of young rats, suggesting that exercise may counteract or delay age-related degenerative changes in parts of the central nervous system [106]. These findings are consistent with previous observations by Skalicky et al., who highlighted how long-term physical activity reduced age-associated functional decline [107].

In the context of traumatic brain injury (TBI), Seo et al. demonstrated that treadmill exercise, lasting ten days, increased Purkinje cell survival and reduced reactive astrocyte formation in the posterior lobules of the cerebellum after head trauma, suggesting a protective role for physical activity [70].

Physical activity shows protective effects even under conditions of hormonal deprivation. In ovariectomized rats, Rauf et al. observed that moderate-intensity intermittent exercise (MIEx) for six weeks could prevent a reduction in Purkinje cell number along with an improvement in cerebellar neurotrophic and neuroprotective markers, as well as cerebellar motor functions in ovariectomized rats. As a result, no interaction was detected between ovariectomy and exercise X [108].

Beyond its role in motor coordination, the cerebellum is increasingly recognized for its involvement in cognitive functions. In a study examining attention deficit/hyperactivity disorder (ADHD), a neurobehavioral disorder characterized by cognitive deficits, Yun et al. evaluated the effects of a 28-day daily treadmill exercise protocol compared to oral methylphenidate (MPH) administration (1 mg/kg daily for 28 consecutive days) as a positive control. The authors demonstrated that both treadmill exercise and MPH treatment significantly improved balance, increased Purkinje cell counts, and simultaneously attenuated astrocytic activation within the cerebellum [109]. Finally, exercise has demonstrated neuroprotective effects on Purkinje cells even in models of chronic cerebral hypoperfusion (CCH) induced by permanent bilateral common carotid artery occlusion (BCCAO). Lee et al. observed that an 8-week treadmill exercise protocol initiated four weeks after birth—prior to the induction of BCCAO at 12 weeks of age—attenuated spatial navigation deficits. This functional improvement was accompanied by a reduction in Purkinje cell loss within the posterior lobe of the cerebellum, as well as a decrease in apoptotic rates and the activation of reactive astrocytes and microglial cells in the cerebellar vermis following CCH. These findings suggest a potential therapeutic role for physical activity in mitigating spatial navigation impairments associated with chronic cerebral hypoperfusion [110].

**Figure 3:**
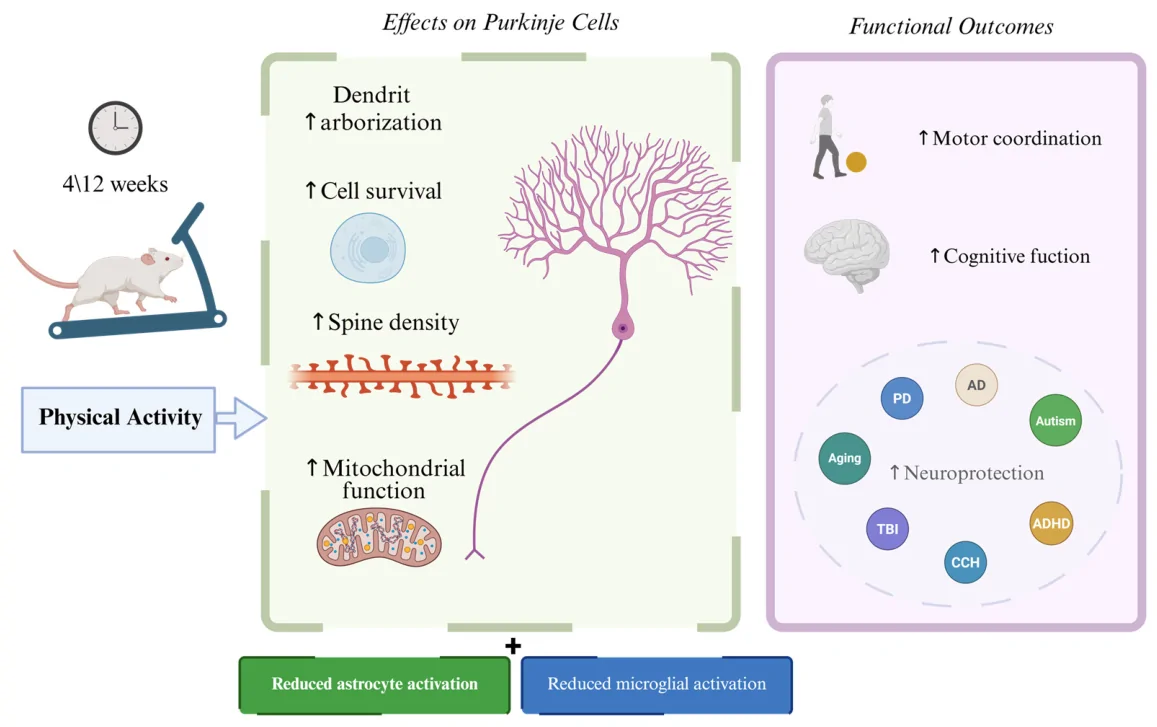
Exercise-induced modulation of Purkinje cell plasticity and survival. Physical activity promotes structural and functional plasticity of Purkinje cells under physiological and pathological conditions. Exercise enhances dendritic arborization, increases spine density, improves mitochondrial function and cell survival. These adaptations contribute to improved motor coordination, cognitive function, and resistance to neurodegenerative processes across multiple disease models. Legend: ↑ increase. Created in https://BioRender.com. Access date: 13 April 2026.

Overall, available experimental studies suggest that physical activity is able to modulate the structure and function of Purkinje cells under different physiological and pathological conditions (Table 1). In particular, exercise protocols of variable duration are associated with increased cell survival, changes in dendritic organization, and improved cerebellar-related motor and cognitive functions (Figure 3). Furthermore, physical activity has shown beneficial effects in several experimental models, including neurodegenerative and neurodevelopmental disorders, traumatic conditions, and hormonal alterations. Although further studies are needed, currently available evidence indicates that physical activity represents a potential modulator of cerebellar plasticity and Purkinje cell function.

## 6. Critical Analysis of Heterogeneity in Exercise Outcomes: Strong vs. Moderate Effects

When evaluating the collective evidence compiled in this review, a noticeable heterogeneity emerges regarding the magnitude of exercise-induced outcomes on Purkinje cells. While certain experimental paradigms report robust neuroprotective effects and massive structural remodeling, others demonstrate only moderate adaptations. A critical analysis of the literature suggests that this divergence is primarily driven by three key methodological variables: Exercise Paradigm and Training Mode: Protocols using forced treadmill running often report stronger, more rapid molecular upregulations (such as sharp increases in BDNF, PACAP, or anti-apoptotic markers) due to the strictly regulated, high-intensity workload imposed on the animals. Conversely, voluntary wheel running typically yields more moderate, gradual structural adaptations, as the total volume and intensity depend entirely on the animal’s spontaneous activity. Pathological Baseline and Insult Severity: The strength of the neuroprotective effect is highly dependent on the baseline condition of the model. In highly aggressive damage models—such as acute traumatic brain injury (TBI), chronic cerebral hypoperfusion (CCH), or direct immunotoxic ablation (OX7-saporin)—the relative gap between sedentary controls (which suffer massive Purkinje cell death) and exercised groups is massive, highlighting a “strong” protective effect. In contrast, in healthy adult models or slowly progressive physiological aging, the degeneration is subtle; hence, exercise produces a more “moderate” maintenance effect rather than a dramatic rescue. Intervention Timing and Window of Vulnerability: The temporal window during which the exercise protocol is introduced represents a crucial determinant. Studies implementing pre-treatment protocols (exercise before the pathological insult, as in some Parkinson’s or ischemia models) or interventions during early postnatal development show significantly stronger outcomes due to the priming of cerebellar resilience. On the other hand, starting the exercise intervention long after the onset of severe neurodegeneration yields only moderate functional recovery, as the extensive loss of Purkinje cells limits the potential for structural remodeling.

## 7. Perspective

In modern society, increasingly characterized by sedentary lifestyles and reduced levels of daily physical activity, identifying effective strategies to maintain brain health has become a major scientific and public health priority. A growing body of evidence indicates that exercise represents one of the simplest, non-pharmacological interventions capable of promoting neuroplasticity and supporting the functional integrity of the brain throughout life. Although traditionally much attention has focused on adaptations of the hippocampus and cortex, only recently has the cerebellum, and particularly Purkinje cells, emerged as a relevant target of exercise-induced neural modulation.

The results analyzed in this review highlight that physical activity exerts structural and functional effects on Purkinje cells under both physiological and pathological conditions. In particular, exercise protocols varying in intensity and duration have been associated with increased Purkinje cell survival, improved dendritic organization, increased synaptic plasticity, and improved motor and cognitive performance in several experimental models. These data suggest that Purkinje cells represent an important cellular target through which physical activity contributes to cerebellar adaptability and neuroprotection. Despite these promising results, most of the available evidence comes from animal studies. Further studies will therefore be needed to clarify the molecular mechanisms underlying the effects of exercise on Purkinje cells and to promote the translation of these findings into the clinical setting. In particular, future research should delve deeper into the role of exercise intensity and duration, potential age- and sex-related differences, and the applicability of physical activity protocols in specific pathological settings, thus better characterizing cerebellar responses to physical activity.

These data suggest that Purkinje cells represent an important cellular target through which physical activity contributes to cerebellar adaptability and neuroprotection.

However, future research must transition from generic exercise paradigms to highly standardized protocols that define the precise dose, intensity, and motor complexity required to optimize Purkinje cell survival. Crucially, a robust translational framework is needed to bridge the gap between rodent data and human clinical applications. Concurrently, investigating sex-specific hormonal interactions and defining age-dependent critical windows will be essential to deliver personalized, precision-medicine interventions across diverse human populations. By combining macro-level structural adjustments and micro-level cellular mechanisms, through the multimodal integration of advanced neuroimaging and peripheral molecular biomarkers, the field can definitively decode how physical movement preserves cerebellar integrity in humans. Ultimately, filling these research gaps will unlock the full potential of exercise as a targeted, non-pharmacological translational strategy to mitigate aging, neurodegenerative disorders, and motor deficits in human patients.

## Figures and Tables

**Table 1:** Effects of exercise on Purkinje cells through physiological and pathological experimental models. Overview of experimental studies reporting structural and functional adaptations of Purkinje cells following different exercise protocols. Legend: ↓decrease; ↑ increase.

Author	Animal Experimental Model	Exercise Type	Exercise Protocol	Reported Effects on Purkinje Cells	Reference
Lee et al.	Rat—Alzheimer’s disease model (Aβ25–35 ICV)	Treadmill exercise	30 min once a day For 4 weeks 4 rpm	↓ Purkinje cell loss; decreased astrocyte activation; ↑ motor coordination	[96]
Jin et al.	Mouse—Alzheimer’s disease model (3xTg-AD)	Treadmill exercise	(12 weeks 5 day/week for a total of 40 min Warm-up (5 min): Initial running at a speed of 5 m/min. Main Exercise Phase (35 min): Continuous running at an increased speed of 8 m/min, corresponding to approximately 40–50% of their ${\mathrm{VO}}_{2}\max$.	↓ extracellular β-amyloid and phosphorylated tau; ↑ mitochondrial function; ↑ Purkinje cell survival	[97]
Lee et al.	Rat—Parkinson’s disease model (rotenone)	Treadmill exercise	once time per a day for 4 weeks 2 m/min during 5 min, at 3 m/min during 5 min, and then at 5 m/min for 20 min with no inclination.	↓ Purkinje cell loss; ↓ astrocytic and microglial activation; ↓ apoptosis; ↑ motor coordination	[98]
Azizi et al.	Rat—Parkinson’s disease model with prenatal/perinatal stress	Aerobic treadmill exercise	8 weeks	↑ Purkinje cell number; attenuation of stress-induced Purkinje cell loss	[99]
Cho et al.	Rat—autism model (valproic acid)	Treadmill exercise	5 times a week, 30 min once a day For 4 weeks 3 m/min for the first 5 min, at a speed of 5 m/min for the next 5 min, and then at a speed of 8 m/min for the last 20 min, with the 0° inclination.	↑ Purkinje cell survival; ↓ astrocyte and microglial activation; ↑ motor function	[100]
Shaban et al.	Mouse—autism model (valproic acid-treated)	Treadmill exercise	6 weeks incremental 30-min treadmill training protocol from (5 to 8 to 11 m/min) intensity at 0% slope for 6 weeks 5 day/week	↑ Purkinje cell survival; ↑ Purkinje cell number; ↓ astrocyte and microglial activation	[101]
Maugeri et al.	Healthy adult rat	Moderate physical activity	5 days a week for 20 min daily For 12 weeks 10–30 m/min	↑ ADNP and β-tubulin III expression in Purkinje cells; ↑ PACAP and PAC1R expression; ↑ DCX co-expression; ↑ neuronal plasticity	[102,103]
González-Burgos et al.	Healthy adult rat	Moderate/intense treadmill exercise	4 weeks: 1 week learning + 3 weeks training for 30 min/day, 5 days/week 15–18 m/min, 0% to 5° slope; preceded by a 7-day shock-assisted learning phase	↑ dendritic spine density; ↑ Purkinje cell synaptic plasticity	[104]
Huang et al.	Young rat	Moderate treadmill exercise	60 min/day, 5 days/week For 8 weeks 12 m/min, increased 3 m/min every 2 week, and reached to 21 m/min	↑ dendritic volume; ↑ spine density; ↑ resistance to OX7-saporin-induced toxicity	[105]
Larsen et al.	Aged rat	Treadmill exercise	two times per day, 5 days a week 20 m/min	↑ Purkinje cell number and soma volume; ↓ age-related degeneration	[106,107]
Seo et al.	Rat—traumatic brain injury model	Treadmill exercise	30 min once a day For 10 days 2 m/min for the first 5 min, 5 m/min for the next 5 min and then 8 m/min for the last 20 min, with the 0° inclination.	↑ Purkinje cell survival; ↓ reactive astrogliosis	[70]
Rauf et al.	Ovariectomized rat	Moderate-intensity intermittent exercise	6 weeks	↑ Purkinje cell number; ↑ neurotrophic markers; ↑ motor performance	[108]
Yun et al.	Rat—ADHD model	Treadmill exercise	30 min once a day, five times a week For 28 days 2 m/min for the first 5 min, at a speed of 5 m/min for the next 5 min, and then at a speed of 8 m/min for the last 20 min, with the 0° inclination.	↑ Purkinje cell number; ↓ astrocyte activation; ↑ balance performance	[109]
Lee et al.	Rat—chronic cerebral hypoperfusion model (BCCAO)	Treadmill exercise	30 min once a day For 8 weeks 2 m/min for the first 5 min, 3 m/min for the following 5 min and 5 m/min for the last 20 min, at 0° inclination	↓ Purkinje cell loss; ↓ glial activation; ↓ apoptosis; ↑ spatial navigation performance	[110]

## Data Availability

No new data were created or analyzed in this study. Data sharing is not applicable to this article.

## Abbreviations

AD	Alzheimer’s disease
ADHD	Attention deficit/hyperactivity disorder
ADNP	Activity-Dependent Neuroprotective Protein
AIS	Axon initial segment
BCCAO	Bilateral common carotid occlusion
CCH	Chronic cerebral hypoperfusion
DCX	Doublecortin
DNC	Deep cerebellar nuclei
ICV	Intracerebroventricular
LTD	Long-term depression
LTP	Long-term potentiation
MIEx	Moderate-intensity intermittent exercise
mPFC	Medial prefrontal cortex
PA	Physical activity
PACAP	Pituitary adenylate cyclase-activating polypeptide
PAG	Periaqueductal gray
PCs	Purkinje cells
PD	Parkinson’s disease
SRM	Social recognition memory
VO	Volume of Oxygen
VOR	Vestibulo-ocular reflex
VPA	Valproic acid
